# Prevalence of hepatitis G virus infection among 67,348 blood donors in mainland China

**DOI:** 10.1186/s12889-019-6948-1

**Published:** 2019-06-03

**Authors:** Taiwu Wang, Juecai Chen, Qi Zhang, Xia Huang, Nanzhen Xie, Jinhai Zhang, Tongjian Cai, Yao Zhang, Hongyan Xiong

**Affiliations:** 1 Department of Epidemiology, College of Preventive Medicine, Army Medical University (Third Military Medical University), Chongqing, 400038 People’s Republic of China; 2 Center for Disease Control and Prevention of Eastern Theater Command, Nanjing, 210002 People’s Republic of China; 3 The First Affiliated Hospital of Chengdu Medical College, Chengdu, 610500 People’s Republic of China; 4 Chongqing Blood Center, Chongqing, 400015 People’s Republic of China; 5 Chongqing General Hospital, Chongqing, 400013 People’s Republic of China

## Abstract

**Background:**

Hepatitis G virus (HGV) infection transmitted from blood donors is a concern in China, as many articles about HGV infection in Chinese blood donors from different provinces have been published. This study aimed to evaluate the overall prevalence of HGV infection in Chinese blood donors and analyse the potential risk of HGV infection through blood transfusion in China.

**Methods:**

We performed a literature search in PubMed, EMBASE, Web of Science, the Chinese BioMedical Literature Database (CBM) and the China National Knowledge Infrastructure (CNKI) up to October 2018 regarding the prevalence of HGV in Chinese blood donors. Eligibility assessment and data extraction were conducted independently by 2 researchers, and meta-analysis was performed to synthesize the data. Heterogeneity was evaluated using Cochran’s Q test and quantified using the *I*^*2*^ statistic. Subgroup analyses were performed to identify the possible sources of heterogeneity. Publication bias was assessed using both funnel plot and Egger’s tests.

**Results:**

A total of 102 studies with 67,348 blood donors published from 1996 to 2016 and covering 26 provinces or municipalities were included for further analyses. The pooled prevalence of HGV was 3.91% (95%CI: 3.18–4.71%) by enzyme immune assay/enzyme linked immunosorbent assay (EIA/ELISA) and 3.25% (95%CI: 2.35–4.26%) by polymerase chain reaction (PCR). The prevalence of HGV may be significantly affected by region, province or municipality and potentially by the paid/voluntary status of the blood donors. No significant difference was found between plasma and full blood donation.

**Conclusions:**

The prevalence of HGV in blood donors from China was similar to that in blood donors from many other countries and higher than that of some other hepatitis viruses, such as hepatitis B virus. The risk of transfusion-transmitted HGV still exists after routine blood donor screening, especially in those patients coinfected with other hepatitis viruses and/or HIV. On the basis of our study, we may suggest adding HGV screening for blood transfusions in mainland China in the future.

**Electronic supplementary material:**

The online version of this article (10.1186/s12889-019-6948-1) contains supplementary material, which is available to authorized users.

## Background

Hepatitis G virus (HGV) or GB virus C (GBV-C) was first discovered and initially identified in 1995, then classified under the Flaviviridae family [1–4], and first detected and reported in 1996 in China [5]. HGV is a newly discovered and enveloped positive-stranded ribonucleic acid (RNA) virus that is structurally and epidemiologically closest to hepatitis C virus (HCV) and may cause acute and chronic hepatitis [6, 7]. HGV replicates primarily in T and B lymphocytes, in peripheral blood mononuclear cells [8], and poorly, if at all, in hepatocytes [9]. HGV may also be able to penetrate the blood-brain barrier and colonize the central nervous system in human immunodeficiency virus (HIV)-infected patients [10]. In addition, HGV could be efficiently transmitted by parenteral routes, such as sexual contact, intra-familiar transmission, intravenous drug use, and exposure to contaminated blood and blood components [11]. Therefore, HGV is highly prevalent among population groups at risk of parenterally transmitted viral agents. Although the pathogenic mechanism of HGV remains unclear [12], previous reports have shown that HGV infection can occur as a single infection or in combination with other viruses such as HCV or HIV [13, 14]. In addition, many studies have shown some rare non-liver diseases to be associated with HGV, such as aplastic anaemia [15, 16], and non-Hodgkin’s lymphoma [17–19].

China placed 48th among 195 countries and territories and selected subnational locations in the healthcare access and quality (HAQ) index, but striking subnational disparities were observed in China, with the HAQ index ranging from 91.5 in Beijing to 48.0 in Tibet [20]. In addition, a portion of the Chinese population is infected with the hepatitis virus, and the hepatitis-related mortality of China was found to be relatively high among the 282 causes of death listed for the 195 countries and territories [21]. Therefore, exploring the prevalence of hepatitis in China is very necessary. HGV, as a subtype of the hepatitis virus, plays an important role in the safety of blood transfusion. Presently, the positive rate of HGV viremia is approximately 1–4% of healthy blood donors in the USA [22, 23], and the proportion of antibodies to the envelope-2 antigen (anti-E2), indicating resolved infection, ranges from 3 to 14% [24]. Overall, the mean prevalence of HGV RNA in blood donors is 4.8% worldwide and varies by ethnicity and location, for example, 1–2% in Saudis [25], 4.5% in Caucasians, 3.4% in Asians, and approximately 17.2% in South Africans and Egyptians [26]. A more serious situation occurs in HIV-infected patients, in which up to 50% of active (HGV viremia) or prior (positive for anti-E2) HGV infection was found [27–29]. Therefore, it is very necessary to estimate the prevalence of HGV in blood donors in China. Although many studies have been published in different provinces or regions, a comprehensive study about the prevalence of HGV in China is still absent. Therefore, we conducted a national systematic review and meta-analysis to assess the prevalence of HGV in blood donors of mainland China.

## Methods

This study was conducted in accordance with the Preferred Reporting Items for Systematic Reviews and Meta-Analyses (PRISMA) [30]. Study searching and selection, quality assessment, and data extraction were performed independently by two researchers (TW and JC). Disagreements were resolved by discussion between the two reviewers and by seeking the opinion of the third author (HX) if necessary.

### Search strategy

An electronic search using keywords for all potential articles was performed through screening PubMed, EMBASE, Web of Science, the Chinese BioMedical Literature Database (CBM), and the China National Knowledge Infrastructure (CNKI). The last search for all databases was performed on Oct 30th, 2018. The keywords used for searching the databases were “hepatitis G virus”, “HGV”, “GB virus C”, “GBV-C”, “blood donors”, “blood centers”, “donation”, “transfusion transmitted disease”, “screening reactive” or “disqualification”, and “China” or “Chinese”. To maximize the output, each keyword was searched individually or in combination. Finally, a total of 580 articles were obtained in the initial search.

### Inclusion and exclusion criteria

Manuscripts included for further analysis had to fulfil all the following criteria: (1) a cross-sectional study; (2) targeted objectives were blood donors from in mainland China; and (3) clear data for calculating the infection rate of HGV and the corresponding detection method. Studies were excluded if they did not fulfil all these criteria.

### Data extraction

The following information of each eligible study was extracted using a data extraction form: first author, year of publication, province, sample size, number of positive cases, diagnostic methods and other information. Data on potential risk factors such as gender, age, sample type, and type of donation (voluntary or paid) were also extracted if present.

### Meta-analysis

A meta-analysis was carried out with R software version 3.4.1 (with the package “meta” version 4.8–4 [31]). Quantum Geographic Information Systems (QGIS) version 2.18 (OSGeo, Beaverton, OR, USA) was used for map construction. Point estimates and 95% confidence intervals (95%CIs) for the prevalence rate of HGV were calculated for each study. To avoid obtaining a confidence interval (CI) outside of a 0–1 range and assigning a large weighting to a study when the proportion becomes too small or too large [32], we calculated prevalence estimates with a variance stabilizing double arcsine transformation [32]. A pooled model was chosen based on the heterogeneity: if obvious heterogeneity existed, a random effects model was adopted; otherwise, a fixed effects model was adopted. Furthermore, sensitivity analysis was performed by converting the pooled results from a random effects model to a fixed effects model or from a fixed effects model to a random effects model. Statistical heterogeneity was evaluated by Cochran’s Q test (with *P* < 0.10 indicating statistically significant heterogeneity) and the *I*^2^ statistic [33]. An *I*^2^ from 0 to 40% was treated as an unimportant heterogeneity, *I*^2^ from 30 to 60% was treated as moderate heterogeneity, *I*^2^ from 50 to 90% was treated as substantial heterogeneity and *I*^2^ from 75 to 100% was treated as considerable heterogeneity [34]. As two detection methods were used in most studies, we performed all analyses stratified by detection methods (EIA/ELISA and PCR). In addition, subgroup analysis was performed based on other potential sources of heterogeneity, such as province, sample type, type of donation (paid or voluntary), regions (Northwest, Southwest, Northeast, South, Central, East and North China), age and gender (if present). Furthermore, a meta-regression was used to investigate any significant difference between/among subgroups and the value of prevalence. Publication bias was examined by funnel plots, and statistical significance was assessed by Egger’s test. In addition, for meta-analysis, we assumed that the included studies contained a random sample from each study population.

## Results

### Characteristics of the eligible studies

**Fig. 1:**
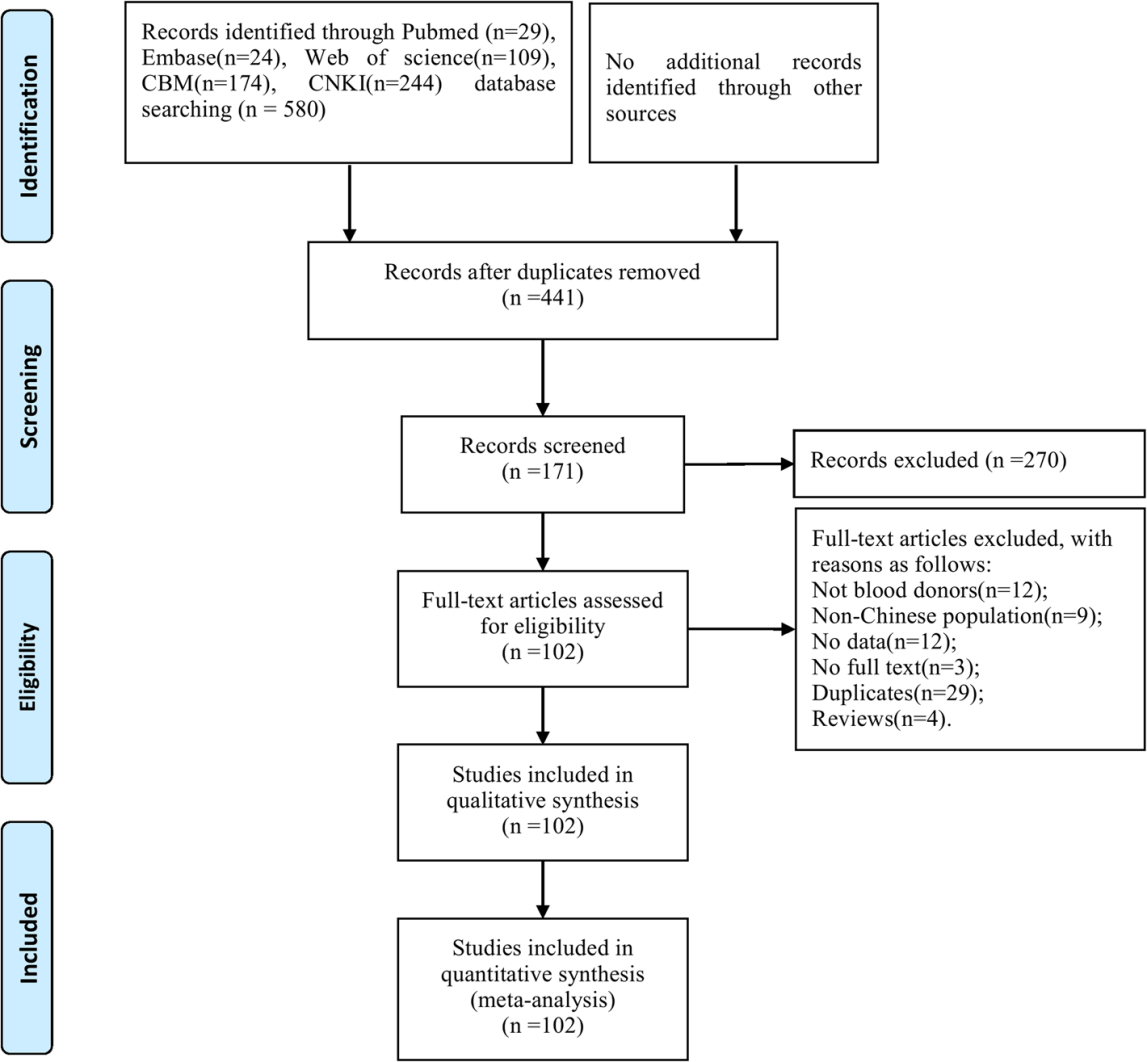
Flowchart describing the study design process

A total of 102 studies meeting the inclusion and exclusion criteria were included for further analysis (Fig. 1). The basic characteristics of the final included studies are shown in Additional file 1: Table S1. These studies were published from 1996 to 2017, covering 26 provinces. As to the detection methods, EIA/ELISA was adopted in 74 studies, while PCR was adopted in 57 studies. The total number of blood donors was 67,348, with a range of 27 to 10,069 per study.

### Overall prevalence of HGV in blood donors

A total of 2066 and 1139 blood samples were shown to have HGV infections, and the overall prevalence of HGV in blood donors was 3.91% (95%CI: 3.18–4.71%) and 3.25% (95%CI: 2.35–4.26%) detected by EIA/ELISA and PCR, respectively. Although the positive rate was slightly higher in the EIA/ELISA detection method, no significant difference was found between the two detection methods (*P* > 0.05).

### Prevalence of HGV from blood donors in relation to risk factors by EIA/ELISA method

**Fig. 2:**
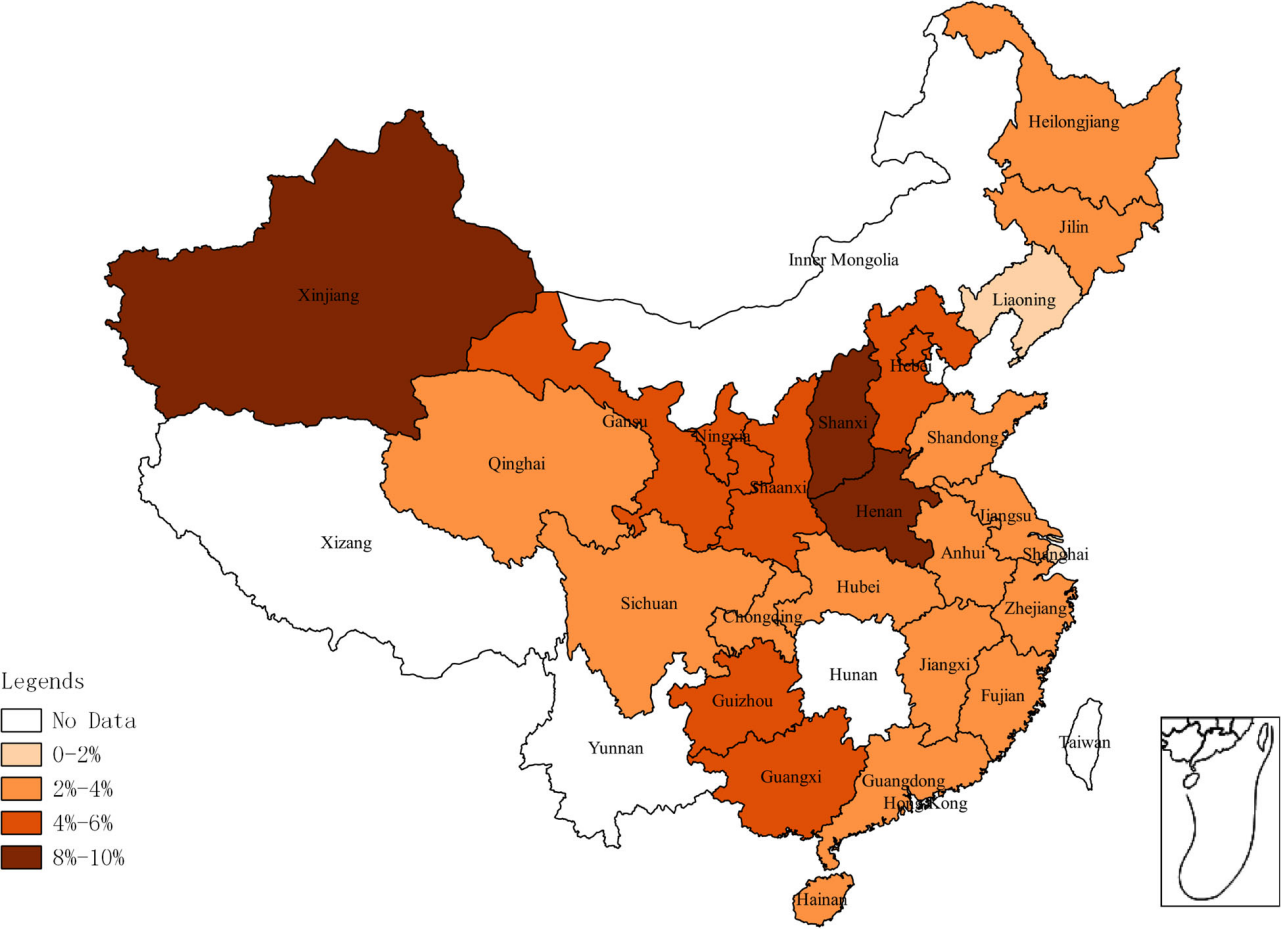
Geographical distribution of HGV prevalence detected by EIA/ELISA

Geographic analysis showed that the highest prevalence of HGV infection by the EIA/ELISA method was in Northwest China (5.68, 95%CI: 4.80–6.63%) and the lowest in Northeast China (4.24, 95%CI: 1.55–4.11%) (Table 1). The prevalence of HGV in blood donors among different provinces is shown in Fig. 2. The highest and lowest prevalence of HGV were found in Henan (9.35, 95%CI: 3.73–17.03%) and Shanghai (1.01, 95%CI: 0.68–1.41%) provinces, respectively. According to meta-regression results of the prevalence in different years, no statistically significant difference in terms of the prevalent trends was found (*P* > 0.05). The lowest and highest prevalence were 0.09% (95% CI: 0.01–0.22%) in 2016 and 13.28% (95% CI: 11.48–15.20%) in 2009 (Fig. 4).

**Table 1:** Seroprevalence for HGV in blood donors associated to risk factors detected by EIA/ELISA

Factors	Categories	No. of studies	No. of blood donors	No. of positivity	Prevalence [95%CI](%)	Heterogeneity		Between-group differences	
						*I* ^*2*^	*P*-value	Q	*P*-value
Region								26.81	0.0002
	Northeast	7	2150	60	2.69[1.55;4.11]	66%	*P* < 0.01		
	East China	32	22,286	544	2.83[2.01;3.76]	92%	*P* < 0.01		
	South China	12	10,800	315	3.84[1.39;7.30]	97%	*P* < 0.01		
	Northwest	13	11,803	637	5.68[4.80;6.63]	62%	*P* < 0.01		
	Central China	9	3958	149	5.37[3.11;8.16]	88%	*P* < 0.01		
	Southwest	6	3133	92	3.21[1.78;5.00]	80%	*P* < 0.01		
	North China	12	6919	270	5.08[2.73;8.08]	95%	*P* < 0.01		
Sample type								0.34	0.56
	Full blood	70	46,022	1376	3.81[3.06;4.63]	94%	*P* < 0.01		
	Plasma	8	15,027	691	4.80[2.30;8.10]	97%	*P* < 0.01		
Type of donation								3.38	0.066
	Voluntary	59	36,232	1033	3.03[2.20;3.97]	95%	*P* < 0.01		
	Paid	32	24,817	1034	4.46[3.47;5.56]	90%	*P* < 0.01		
Sex								0.43	0.51
	Male	10	14,031	469	3.06[1.22;5.63]	98%	*P* < 0.01		
	Female	10	10,310	390	4.10[2.01;6.85]	96%	*P* < 0.01		
Age								3.48	0.32
	21–30	3	530	15	2.13[0.88;3.76]	0	*P* = 0.60		
	31–40	3	597	15	2.10[0.88;3.70]	10%	*P* = 0.33		
	41–50	3	434	19	4.03[1.44;7.60]	42%	*P* = 0.18		
	51-	1	10	1	10.0[0.0;38.09]	0	–

Other factors that may affect the prevalence of HGV were analysed. The pooled estimates by potential risk factors associated with HGV infection in blood donors are presented in detail in Table 1. Of all the factors analysed in our study, the prevalence of HGV infection in different regions was significantly different (*P* < 0.01). In addition, the prevalence of HGV detected by the EIA/ELISA method in paid blood donors (4.46, 95% CI: 3.47–5.56%) was higher than that in voluntary blood donors (3.03, 95% CI: 2.20–3.97%), with a *P* value of 0.066.

### Prevalence of HGV from blood donors in relation to risk factors by PCR

**Fig. 3:**
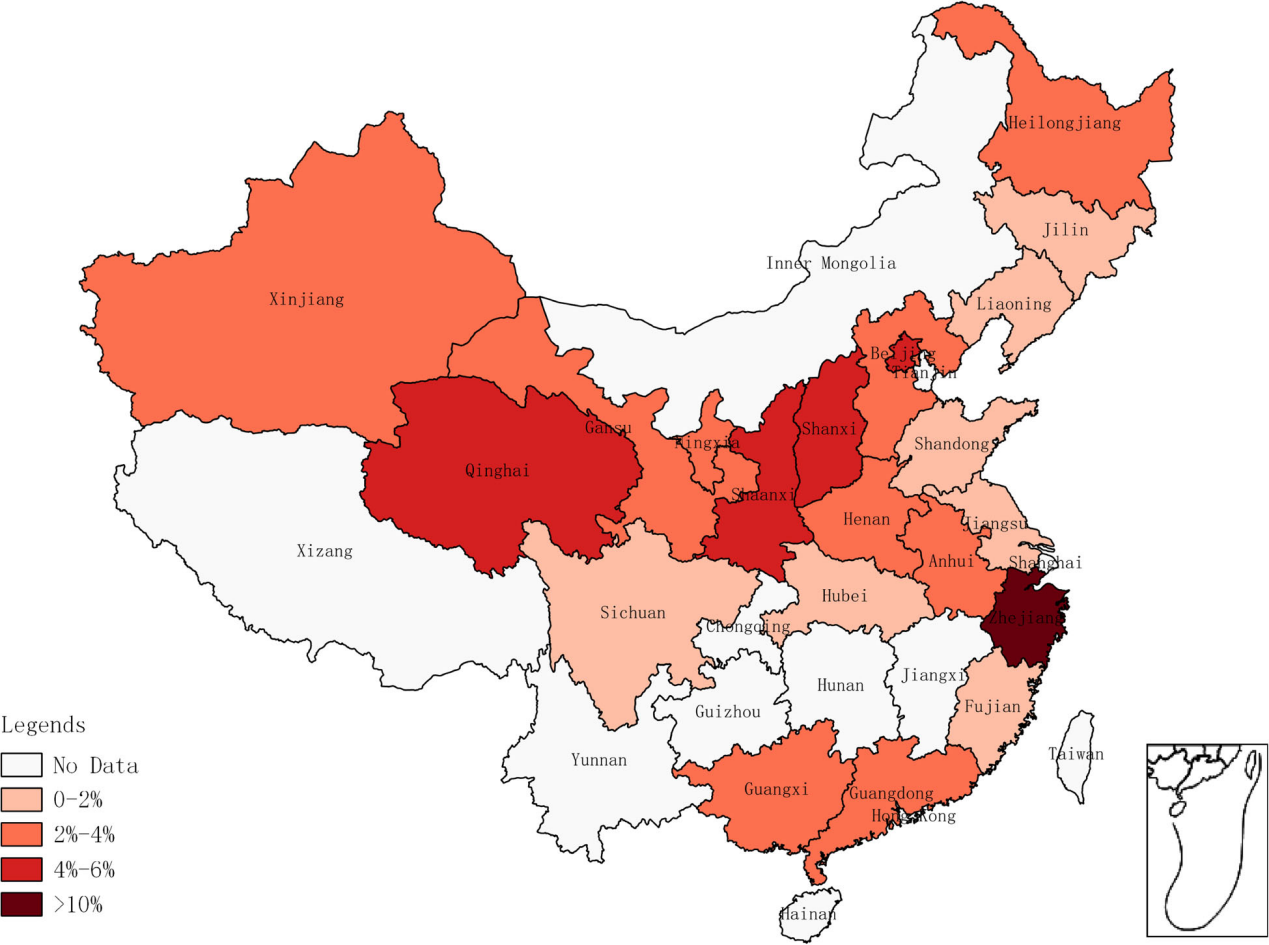
Geographical distribution of HGV prevalence detected by PCR

**Fig. 4:**
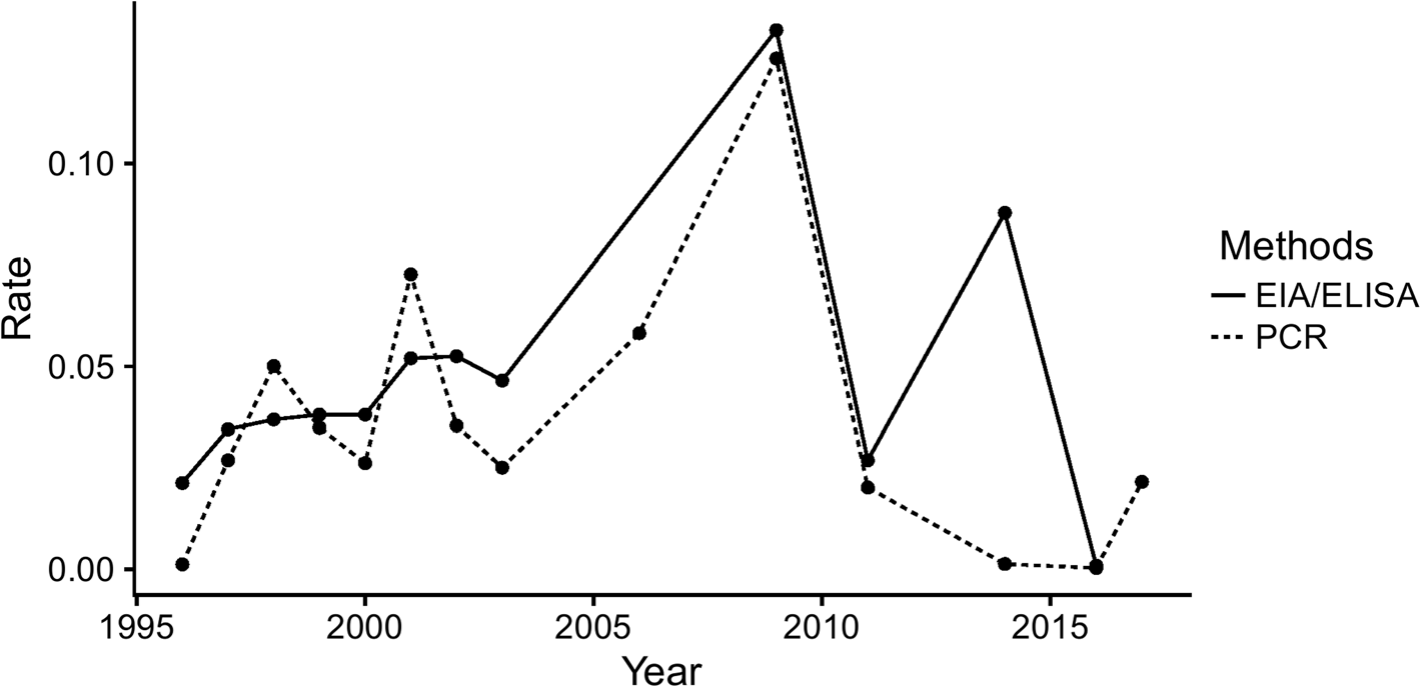
Prevalence of HGV in each year grouped by detection methods

The prevalence of HGV infection by PCR was similar to EIA/ELISA. The highest prevalence of HGV infection by the PCR detection method was in North China (4.08, 95% CI: 2.26–6.34%) and the lowest in Northeast China (1.49, 95% CI: 0.90–2.22%) (Table 2). The prevalence of HGV in blood donors among different provinces is shown in Fig. 3. The highest and lowest prevalence of HGV were found in Zhejiang (15.21, 95% CI: 11.70–19.09%) and Jilin (0.77, 95%CI: 0.01–2.31%) provinces, respectively. According to the meta-regression results of the prevalence in different years, no statistically significant difference in terms of the prevalent trends was found (*P* > 0.05). The lowest and highest prevalence was 0.03% (95% CI: 0.00–0.12%) in 2016 and 12.58% (95% CI: 10.82–14.45%) in 2009 (Fig. 4).

**Table 2:** Seroprevalence for HGV in blood donors associated to risk factors detected by PCR

Factors	Categories	No. of studies	No. of blood donors	No. of positivity	Prevalence [95%CI](%)	Heterogeneity		Between-group differences	
						*I* ^*2*^	*P*-value	Q	*P*-value
Region								23.50	0.0006
	East China	22	10,070	205	2.97[1.50;4.85]	95%	*P* < 0.01		
	South China	10	9071	226	3.40[0.55;8.17]	98%	*P* < 0.01		
	Central China	9	2164	53	2.60[0.92;4.95]	84%	*P* < 0.01		
	North China	18	7436	210	4.08[2.26;6.34]	93%	*P* < 0.01		
	Northeast	4	1545	24	1.49[0.90;2.22]	8%	*P* = 0.35		
	Southwest	1	119	2	1.68[0.03;5.00]	–	–		
	Northwest	8	11,204	418	3.78[3.20;4.41]	49%	*P* = 0.06		
Sample type								0.20	0.65
	Full blood	51	24,986	697	3.35[2.31;4.56]	95%	*P* < 0.01		
	Plasma	8	14,182	442	2.94[1.06;5.63]	97%	*P* < 0.01		
Type of donation								7.09	0.008
	Voluntary	40	18,666	472	2.26[1.24;3.53]	95%	*P* < 0.01		
	Paid	28	20,502	667	4.62[3.34;6.07]	92%	*P* < 0.01

The pooled estimates by potential risk factors associated with HGV infection in blood donors are presented in detail in Table 2. The prevalence of HGV infection in different regions was significantly different (*P* < 0.01). In addition, whether the blood donors were paid or not affected the prevalence significantly (*P* = 0.008), with results of 2.26% (95% CI: 1.24–3.53%) in voluntary and 4.62% (95% CI: 3.34–6.0%) in paid blood donors.

**Fig. 5:**
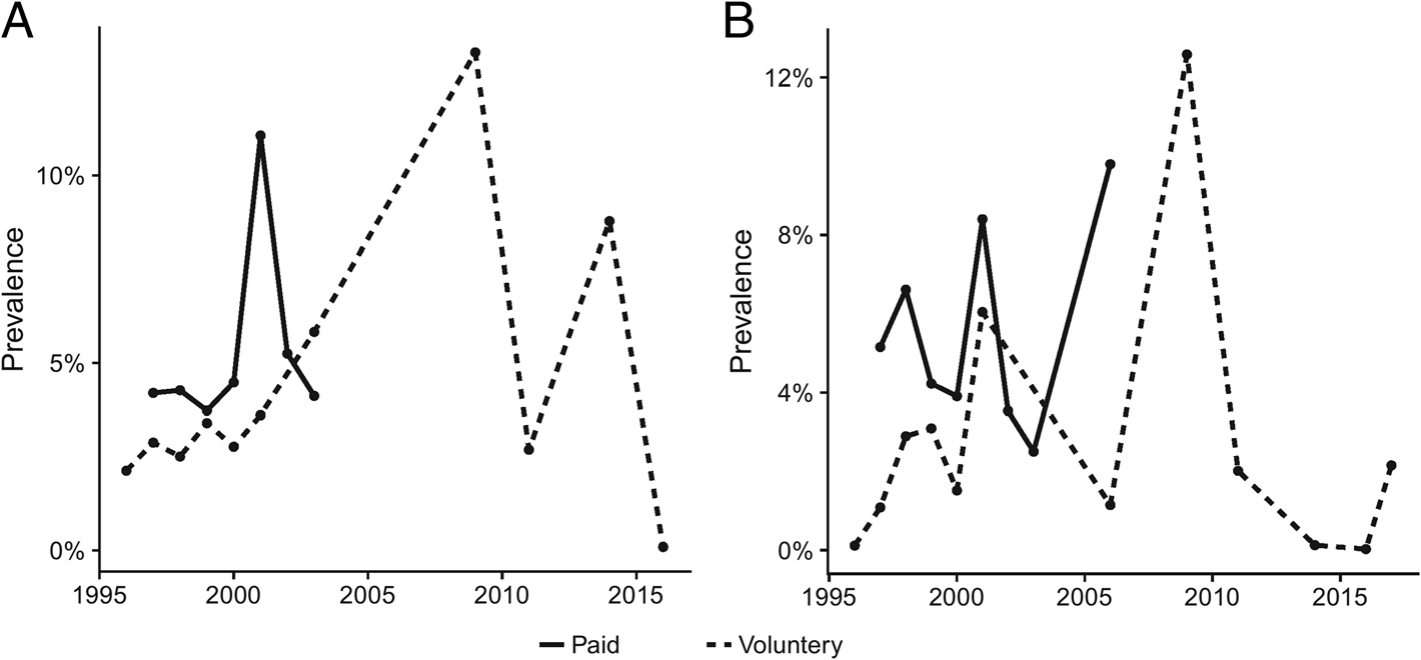
Prevalence of HGV in each year grouped by type of donation. (**a**: detected by EIA/ELISA, **b**: detected by PCR)

Given the large differences between paid and volunteer blood donors in both EIA/ELISA and PCR, we analysed the prevalence of HGV separately in paid and volunteer blood donors as a function of publication year. As shown in Fig. 5, the prevalence of HGV in paid blood donors was always higher than in volunteer blood donors at the same time. However, some peaks still existed, especially in 2009.

### Publication bias and sensitivity tests

**Fig. 6:**
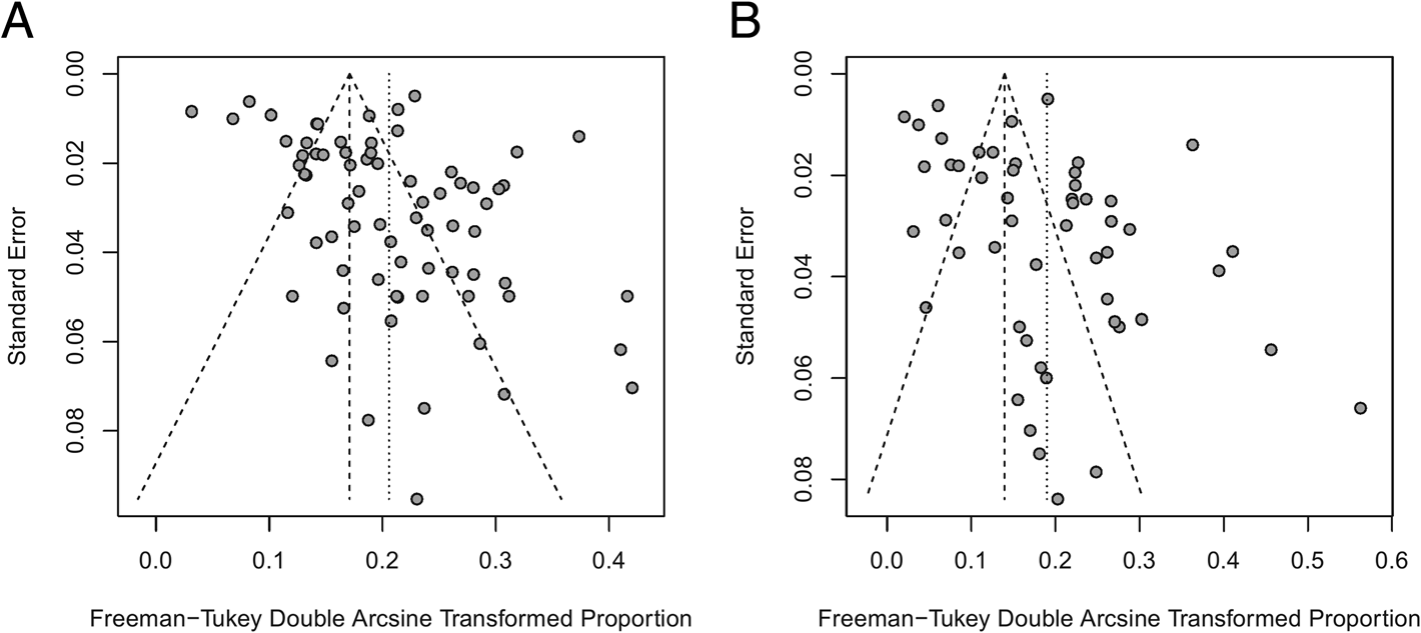
Funnel plot of HGV prevalence by detection methods, showing potential publication bias. (**a**: detected by EIA/ELISA, **b**: detected by PCR)

A funnel plot and Egger’s test were employed to examine publication bias. As shown in Fig. 6, the funnel plot indicates that publication bias may exist, which was also confirmed by Egger’s test (*P* = 0.01 for both EIA/ELISA and PCR). Sensitivity analysis was conducted for the pooled results by converting the pooled model (from a random effects model to a fixed effects model). The results exhibited no large differences in proportion and 95%CIs before and after pooling, indicating stability in the pooled results.

## Discussion

Since HGV was discovered in 1995, many studies have been conducted to investigate the prevalence of HGV in different populations, including blood donors. However, no systematic review on HGV prevalence among blood donors in mainland China has performed. As HGV can be transmitted by blood, it should be of great concern in the safety of blood transfusion. In this study, 102 studies were included to investigate the prevalence of HGV in 67,348 blood donors across mainland China. To our knowledge, this is the first report to estimate the national level of HGV prevalence among blood donors in mainland China, which could be important to public health surveillance and disease prevention and control policies.

A diagnosis of HGV is mainly achieved according to PCR and EIA/ELISA results in biological samples, with the PCR technique possibly being more valuable for detecting current infections [35]. In our study, both PCR and EIA/ELISA were adopted. The overall prevalence of HGV in blood donors in mainland China was 3.91% (95% CI, 3.18–4.71%) by EIA/ELISA and 3.25% (95%CI: 2.35–4.26%) by PCR with obvious heterogeneity. Although the prevalence by EIA/ELISA was slightly higher than by PCR, no significant difference was found. In addition, as the total sample size seems sufficiently large, this heterogeneity may be due to confounding factors, such as region, type of donation, and other factors (for example, ALT level [36]). Overall, the prevalence of HGV in China was similar to that in the USA, Asia, France, and among Caucasians, and lower than that in South Africa and Egypt [22, 23, 26, 37].

Through our research, HGV is widely distributed in blood donors throughout mainland China. As reported before [38], the pooled prevalence of HBsAg and OBI among donors was 1.085 and 0.094%, respectively, indicating that the prevalence of HGV was higher than that of HBV in blood donors. In addition, a previous study reported that the pooled rates of anti-HEV IgM- and IgG-positive donations were 1.09 and 30%, respectively, and anti-HEV IgM- and IgG-positive rates were higher in the Southwest region compared to those in other regions of China [36, 39]. Regarding HCV, the pooled prevalence among blood donors in mainland China was 8.68%, and the epidemic situation was more serious in North and Central China, especially in Henan and Hebei provinces [40]. Therefore, we could see that the prevalence of HGV was relatively high compared with other hepatitis viruses. At the same time, the region could affect the prevalence of all these hepatitis viruses. However, as mentioned above, the prevalent regions of different hepatitis viruses were different. HGV and HCV are higher in North and Central China, while HEV is higher in Southwest China and HBV is higher in developed areas (East China). In addition, the presence of a co-infection virus may also affect the detectable rate [27].

We also found that the type of donation may significantly affect the prevalence of HGV. When the Blood Donation Law was implemented in 1998 in China, the Chinese government banned paid blood donations for clinical use and encouraged 100% usage of voluntary blood donations. However, as reported in our study, the prevalence increased for a few years and then decreased. We found only one study reported in 2009, the year of highest prevalence, which may not estimate the overall prevalence exactly; this study was performed in Henan, where there was a more serious problem regarding paid blood donations than in other provinces. Therefore, the prevalence in 2009 may be overrated. Overall, the prevalence of HGV in blood donors has decreased since the implementation of the Blood Donation Law, especially after 2009. In addition, plasma donors were found to have a relatively higher prevalence of HCV infection than whole blood donors [40], but no difference was found between plasma and full blood donation in our study for HGV prevalence. This may be attributed to the different characteristics of different hepatitis viruses and many other confounding factors, as obvious heterogeneity still exists in our study.

Despite the decreasing trend of HGV prevalence after its peak in 2009, the overall prevalence of HGV has been relatively high compared with other hepatitis viruses in some provinces. Therefore, the risk of transfusion-transmitted HGV still exists. On the basis of our study, we may suggest adding HGV screening for blood transfusions in mainland China in the future.

## Limitations

The meta-analysis results presented here are subject to many limitations: 1) All studies used a cross-sectional observational study design; 2) Most of the literature included in this study was in Chinese language and very few in English; 3) The reagents used for HGV screening testing were from different manufacturers or different generations and were performed by different operators, which may lead to heterogeneity; 4) The articles included in this study were primarily published ten to twenty years ago, with only 7 papers published in the last ten years, demonstrating that less attention has been paid to HGV infection in blood donors lately; 5) Only a few confounding factors were analysed for high heterogeneity, but the primary cause is still unknown; 6) Publication bias still exists.

## Conclusions

The prevalence of HGV among blood donors in mainland China was similar to that in many other countries and higher than that of other hepatitis viruses. Therefore, qualified donations after routine blood donor screening may still carry a potential risk for transmitting HGV. Despite the decreasing trend of HGV prevalence after its peak in 2009, it is urgent to make efficient measures to prevent HGV transmission from blood donors. Our study provides an overall prevalence of HGV in blood donors for further management, and on the basis of our study, we may suggest adding HGV screening for blood transfusions in the future.

## Additional File

Additional file 1: **Table S1.** Baseline characteristics of the included studies. (DOC 409 kb)

## Abbreviations

CI: Confidence interval
EIA/ELISA: Enzyme immune assay/enzyme linked immunosorbent assay
HGV: Hepatitis G virus
HIV: Human immunodeficiency virus
PCR: Polymerase chain reaction
