# Supplementary material for: Prevalence of hepatitis G virus infection among 67,348 blood donors in mainland China
Source: BMC Public Health. 2019 Jun 3;19:685. doi: 10.1186/s12889-019-6948-1 (PMC6547458; doi:10.1186/s12889-019-6948-1)
Supplement: Supplementary file 1 — Table S1. Baseline characteristics of the included studies. (DOC 409 kb) [file 12889_2019_6948_MOESM1_ESM.doc]

Supplementary table 1 Baseline characteristics of included studies

| Author | Year | Province | Region | Population | Sample type | EIA/ELISA | PCR | Total |
| --- | --- | --- | --- | --- | --- | --- | --- | --- |
| Cao et al. | 1996 | Beijing | North China | Voluntary blood donors | Full blood | 16 |  | 302 |
| Wang et al. | 1996 | Hebei | North China | Voluntary blood donors | Plasma | 11 | 3 | 2500 |
| Ren et al. | 1997 | Shanxi | North China | Paid blood donors | Full blood |  | 1 | 50 |
| Ma et al. | 1997 | Jiangsu | East China | Voluntary blood donors | Full blood | 3 | 0 | 258 |
| Wang et al. | 1997 | Beijing | North China | Paid blood donors，Voluntary blood donors | Full blood |  | 19 | 410 |
| Zhou et al. | 1997 | Guangdong | South China | Paid blood donors | Full blood |  | 9 | 106 |
| Wang et al. | 1997 | Beijing | North China | Paid blood donors | Full blood |  | 21 | 265 |
| He et al. | 1997 | Beijing | North China | Paid blood donors，Voluntary blood donors | Full blood | 17 |  | 600 |
| Wang et al. | 1997 | Jilin, Guizhou, Hebei, Anhui | Northeast, Southwest, North China, East China | Voluntary blood donors | Plasma | 69 | 6 | 1550 |
| Chen et al. | 1997 | Hebei | North China | Voluntary blood donors | Plasma | 11 |  | 203 |
| Qiu et al. | 1997 | Guangdong | South China | Voluntary blood donors | Full blood | 28 |  | 1080 |
| Feng et al. | 1997 | Hainan | South China | Paid blood donors | Full blood | 3 |  | 81 |
| Wang et al. | 1997 | Jiangxi | East China | Paid blood donors | Full blood | 8 |  | 219 |
| He et al. | 1997 | Beijing | North China | Paid blood donors | Full blood | 37 | 16 | 1049 |
| Wang et al. | 1997 | Guangxi, Fujian, Sichuan, Hubei, Jiangsu, Gansu, Shandong, Beijing, Heilongjiang, Jilin | South China, East China, Southwest, Central China, East China, Northwest, East China, North China, Northeast, Northeast | Voluntary blood donors | Full blood | 100 | 62 | 2870 |
| Bao et al. | 1997 | Xinjiang | Northwest | Paid blood donors，Voluntary blood donors | Full blood | 10 |  | 65 |
| Li et al. | 1997 | Shaanxi | Northwest | Voluntary blood donors | Full blood | 7 |  | 100 |
| Ma et al. | 1997 | Liaoning | Northeast | Voluntary blood donors | Full blood | 1 |  | 100 |
| Lei et al. | 1998 | Zhejiang | East China | Voluntary blood donors | Full blood | 12 |  | 749 |
| Ren et al. | 1998 | Beijing, Shanxi, Hebei | North China | Paid blood donors | Plasma |  | 12 | 279 |
| Ru et al. | 1998 | Guangxi | South China | Paid blood donors | Full blood | 6 |  | 140 |
| Yang et al. | 1998 | Sichuan | Southwest | Paid blood donors | Full blood | 4 |  | 48 |
| Zhang et al. | 1998 | Fujian | East China | Voluntary blood donors | Full blood | 4 |  | 187 |
| Bao et al. | 1998 | Xinjiang | Northwest | Paid blood donors，Voluntary blood donors | Full blood | 9 | 2 | 100 |
| Wei et al. | 1998 | Hubei | Central China | Voluntary blood donors | Full blood | 2 | 2 | 90 |
| Xu et al. | 1998 | Henan | Central China | Voluntary blood donors | Full blood | 15 |  | 200 |
| Fu et al. | 1998 | Guangdong | South China | Paid blood donors | Full blood |  | 9 | 106 |
| Yang et al. | 1998 | Jiangsu | East China | Paid blood donors，Voluntary blood donors | Full blood | 4 |  | 99 |
| Song et al. | 1998 | Anhui | East China | Paid blood donors | Full blood | 7 |  | 131 |
| Ling et al. | 1998 | Hebei | North China | Voluntary blood donors | Full blood，Plasma | 80 | 41 | 819 |
| Duan et al. | 1998 | Chongqing | Southwest | Voluntary blood donors | Full blood | 23 |  | 685 |
| Liu et al. | 1998 | Hebei | North China | Voluntary blood donors | Full blood | 10 |  | 113 |
| Jiang et al. | 1998 | Guangdong | South China | Paid blood donors，Voluntary blood donors | Full blood | 43 | 23 | 6426 |
| Feng et al. | 1998 | Shandong | East China | Voluntary blood donors | Full blood | 40 |  | 2025 |
| Deng et al. | 1998 | Jiangxi | East China | Paid blood donors | Full blood | 8 |  | 485 |
| Mao et al. | 1998 | Zhejiang | East China | Paid blood donors，Voluntary blood donors | Full blood | 23 |  | 620 |
| Cong et al. | 1998 | Henan | Central China | Paid blood donors | Full blood |  | 16 | 57 |
| Yang et al. | 1998 | Zhejiang | East China | Paid blood donors | Full blood | 1 |  | 41 |
| Wang et al. | 1998 | Zhejiang | East China | Voluntary blood donors | Full blood | 3 |  | 174 |
| Zhao et al. | 1999 | Hubei | Central China | Voluntary blood donors | Full blood | 40 |  | 1997 |
| Chen et al. | 1999 | Shandong | East China | Voluntary blood donors | Full blood | 11 |  | 361 |
| Song et al. | 1999 | Anhui | East China | Voluntary blood donors | Full blood | 29 | 18 | 385 |
| Huang et al. | 1999 | Guangxi | South China | Voluntary blood donors | Full blood | 5 |  | 68 |
| Liu et al. | 1999 | Shanghai | East China | Voluntary blood donors | Full blood | 30 |  | 2968 |
| Yao et al. | 1999 | Guangdong | South China | Voluntary blood donors | Full blood |  | 2 | 69 |
| Zhang et al. | 1999 | Shandong | East China | Paid blood donors | Full blood | 21 |  | 348 |
| Chen et al. | 1999 | Shandong | East China | Voluntary blood donors | Full blood | 11 |  | 361 |
| Song et al. | 1999 | Anhui | East China | Paid blood donors | Full blood |  | 22 | 409 |
| Zhou et al. | 1999 | Anhui | East China | Paid blood donors，Voluntary blood donors | Full blood | 177 |  | 3948 |
| Ye et al. | 1999 | Anhui | East China | Paid blood donors | Full blood | 18 | 12 | 1050 |
| Shen et al. | 1999 | Henan | Central China | Voluntary blood donors | Full blood | 16 |  | 100 |
| Li et al. | 1999 | Guangdong | South China | Voluntary blood donors | Full blood |  | 2 | 69 |
| Wang et al. | 1999 | Hebei | North China | Voluntary blood donors | Full blood | 5 | 7 | 100 |
| Yang et al. | 1999 | Jiangsu | East China | Paid blood donors，Voluntary blood donors | Full blood | 4 |  | 100 |
| Yang et al. | 1999 | Hubei | Central China | Voluntary blood donors | Full blood |  | 2 | 74 |
| Zhao et al. | 1999 | Henan | Central China | Voluntary blood donors | Full blood |  | 1 | 300 |
| Liu et al. | 1999 | Jiangsu | East China | Voluntary blood donors | Full blood |  | 1 | 60 |
| Wu et al. | 1999 | Shandong | East China | Paid blood donors，Voluntary blood donors | Full blood，Plasma | 15 | 4 | 780 |
| Pan et al. | 1999 | Jiangsu | East China | Paid blood donors | Full blood |  | 2 | 40 |
| Wang et al. | 1999 | Beijing | North China | Paid blood donors，Voluntary blood donors | Full blood |  | 16 | 84 |
| Wang et al. | 1999 | Shandong | East China | Voluntary blood donors | Full blood |  | 7 | 104 |
| Ling et al. | 2000 | Anhui | East China | Paid blood donors，Voluntary blood donors | Full blood | 22 |  | 808 |
| Zhen et al. | 2000 | Guangdong | South China | Voluntary blood donors | Full blood | 4 | 0 | 117 |
| Tian et al. | 2000 | Hubei | Central China | Voluntary blood donors | Full blood | 29 | 8 | 417 |
| Liu et al. | 2000 | Chongqing | Southwest | Paid blood donors | Full blood | 23 |  | 685 |
| Jia et al. | 2000 | Liaoning | Northeast | Paid blood donors，Voluntary blood donors | Full blood | 9 | 7 | 597 |
| Zhang et al. | 2000 | Shandong | East China | Voluntary blood donors | Full blood | 1 |  | 60 |
| Yang et al. | 2000 | Shandong | East China | Paid blood donors，Voluntary blood donors | Full blood，Plasma | 16 | 5 | 762 |
| Li et al. | 2000 | Guangxi | South China | Voluntary blood donors | Full blood | 8 |  | 496 |
| Xing et al. | 2000 | Henan | Central China | Voluntary blood donors | Full blood |  | 1 | 200 |
| Zhou et al. | 2000 | Jiangxi | East China | Voluntary blood donors | Full blood | 14 |  | 215 |
| Huang et al. | 2000 | Guangxi | South China | Voluntary blood donors | Full blood | 6 | 3 | 213 |
| Mei et al. | 2000 | Henan | Central China | Paid blood donors，Voluntary blood donors | Full blood | 29 | 18 | 800 |
| Li et al. | 2000 | Guangdong | South China | Voluntary blood donors | Full blood | 1 |  | 27 |
| Cao et al. | 2000 | Shaanxi, Qinghai, Xinjiang | Northwest | Voluntary blood donors | Full blood | 21 |  | 432 |
| Song et al. | 2000 | Anhui | East China | Paid blood donors，Voluntary blood donors | Full blood |  | 32 | 662 |
| Li et al. | 2000 | Anhui | East China | Paid blood donors | Full blood |  | 13 | 201 |
| Li et al. | 2000 | Hubei | Central China | Paid blood donors，Voluntary blood donors | Full blood | 3 |  | 128 |
| Li et al. | 2000 | Shaanxi | Northwest | Voluntary blood donors | Full blood |  | 27 | 397 |
| Wang et al. | 2000 | Jiangsu | East China | Voluntary blood donors | Full blood | 8 | 8 | 126 |
| Zhang et al. | 2000 | Shanxi | North China | Paid blood donors，Voluntary blood donors | Full blood | 24 | 20 | 295 |
| Li et al. | 2000 | Xinjiang | Northwest | Paid blood donors | Full blood | 36 |  | 399 |
| Wang et al. | 2000 | Shandong | East China | Paid blood donors | Full blood |  | 1 | 44 |
| Feng et al. | 2001 | Henan | Central China | Voluntary blood donors | Full blood | 8 | 1 | 50 |
| Yang et al. | 2001 | Zhejiang | East China | Voluntary blood donors | Full blood |  | 32 | 203 |
| Liu et al. | 2001 | Sichuan | Southwest | Voluntary blood donors | Full blood | 14 |  | 1104 |
| Cheng et al. | 2001 | Xinjiang | Northwest | Paid blood donors，Voluntary blood donors | Full blood | 34 | 25 | 518 |
| Li et al. | 2001 | Beijing | North China | Voluntary blood donors | Plasma | 7 | 5 | 176 |
| Liu et al. | 2001 | Zhejiang | East China | Voluntary blood donors | Full blood |  | 24 | 165 |
| Li et al. | 2002 | Beijing, Shanxi, Hebei | North China | Paid blood donors | Plasma |  | 12 | 279 |
| Han et al. | 2002 | Jilin | Northeast | Paid blood donors | Full blood | 9 |  | 123 |
| Xi et al. | 2002 | Gansu, Shaanxi, Ningxia, Qinghai | Northwest | Paid blood donors | Plasma | 516 | 362 | 10069 |
| Wang et al. | 2003 | Shandong | East China | Paid blood donors，Voluntary blood donors | Full blood | 12 |  | 240 |
| Bai et al. | 2003 | Shandong | East China | Paid blood donors | Full blood | 2 | 1 | 44 |
| Wang et al. | 2003 | Jiangsu | East China | Paid blood donors | Full blood |  | 1 | 35 |
| Wu et al. | 2006 | Guangdong | South China | Paid blood donors，Voluntary blood donors | Full blood |  | 11 | 189 |
| Huang et al. | 2009 | Guangxi | South China | Voluntary blood donors | Full blood | 170 | 161 | 1280 |
| Jia et al. | 2011 | Liaoning | Northeast | Voluntary blood donors | Full blood | 8 | 6 | 297 |
| Xiao et al. | 2014 | Beijing, Guangdong, Jiangsu, Shanxi, Sichuan, Jiangsu | no detail information | Voluntary blood donors | Full blood |  | 1 | 748 |
| Yu et al. | 2014 | Guangdong | South China | Voluntary blood donors | Full blood | 33 |  | 376 |
| Jin et al. | 2016 | Jiangsu | East China | Voluntary blood donors | Full blood | 3 | 1 | 3520 |
| Shao et al. | 2017 | Beijing | North China | Voluntary blood donors | Full blood |  | 15 | 694 |

EIA/ELISA: enzyme immune assay/enzyme linked immunosorbent assay; PCR: Polymerase Chain Reaction

**References**

1. Cao Y, Cheng Y, Zhang J, Guo R, Cheng B, Jiang P, He J, Tao L, Qiao X: **Detection of anti-hepatic hepatitis virus antibody in serum of some people in Beijing area**. *Chinese Journal of Experimental and Clinical Virology* 1996, **3**(10):279-280.

2. Wang X, Zhuang H, Li H, Fan J, Qi Z, Liu G: **Detection of hepatitis C virus (gbv-c) infection in Chinese strains and determination of partial nucleic acid sequence**. *Chinese Journal of Microbiology and Immunology* 1996, **16**(4):263-266.

3. Ren F, Li H, Zhao H, Wang Y: **Results of rt-pcr detection of hepatic hepatitis virus in blood of 100 individual blood donors**. *Chinese Journal of Blood Transfusion* 1997, **10**(4):208.

4. Ma D, Guo N, Xu N: **Detection of serum anti-HGV-IgG in 972 samples**. *Chinese Journal of Clinical Laboratory Science* 1997(6):350-351.

5. Wang Y, Chen HS, Fan MH, Liu HL, An P, Sawada N, Tanaka T, Tsuda F, Okamoto H: **Infection with GB virus C and hepatitis C virus in hemodialysis patients and blood donors in Beijing**. *Journal of medical virology* 1997, **52**(1):26-30.

6. Zhou B, Ma W, Wang H, Fu Y, Xu L, Lu L, Jiang F, Peng W: **[Investigation on hepatitis G virus (HGV) infection among different populations in Shenzhen]**. *Zhonghua shi yan he lin chuang bing du xue za zhi = Zhonghua shiyan he linchuang bingduxue zazhi = Chinese journal of experimental and clinical virology* 1997, **11**(4):348-351.

7. Wang HL, Jin DY: **Prevalence and genotype of hepatitis G virus in Chinese professional blood donors and hepatitis patients**. *The Journal of infectious diseases* 1997, **175**(5):1229-1233.

8. He H, Mao P, Han J, Bai Y, Ju L, Zhu L, Hong S: **[Primary analysis of the situation of hepatitis G virus infection in different groups of people]**. *Zhonghua shi yan he lin chuang bing du xue za zhi = Zhonghua shiyan he linchuang bingduxue zazhi = Chinese journal of experimental and clinical virology* 1997, **11**(4):382-383.

9. Wang X, Hao J, Zhuang H: **[Studies on GBV-C infection in blood donors in four provinces of China]**. *Zhonghua yu fang yi xue za zhi [Chinese journal of preventive medicine]* 1997, **31**(6):349-351.

10. Chen z, Liu C, Yang Y, Wang X, Li H: **Retrospective study on hepatic hepatitis virus infection**. *Disease Surveillance* 1997, **12**(6):211-212.

11. Qiu Y, Yang C: **Investigation on Hepatitis G Virus Antibodies of 1080 Blood Donors in Guangzhou**. *Journal of First Military Medical University* 1997, **17**(4):324.

12. Feng Q, He Q, Lu X, Ruan G, You M, Li G, Li Y: **Serum Hepatitis G Antibodies in Different Populations in Haikou**. *Hainan Medical Journal* 1997, **8**(3):215-216.

13. Wang K, Li XJ, Yin HL, Zhou HB, Chen FF, Wang H, Ma HH: **[Expression of ID3 protein in prostate cancer and its clinicopathological significance]**. *Zhonghua nan ke xue = National journal of andrology* 2011, **17**(5):410-413.

14. He H, Mao P, Hong S, Ju L, Hu Y, Bai Y, Yang J: **Study on Hepatitis G Virus Infection of 1049 Professional Blood Donors in Some Areas of China**. *Chinese Journal of Public Health* 1997, **13**(5):266-267.

15. Wang Y, Zhuang H, Wu X, Chen Z, Hao J, Pan H, Wang X, Xue D, Qi Z, Harrison T *et al*: **Investigation on Hepatitis G Virus Infection in Blood Donors in Some Areas of China**. *Chinese Journal of Public Health* 1997, **13**(11):657-658.

16. Bao Y, Xun H, Niu H, Li G: **Results of G-hepatitis antibody test in some people in Urumqi**. *Journal of Xinjiang Medical University* 1997(3):57-58.

17. Li M, Xu Z, Xie X, Wang Q, Tian R: **A preliminary survey of hepatic infection in blood donors in Xi'an area**. *Shaanxi Journal of Medical Laboratory Sciences* 1997, **12**(2):51-52.

18. Ma L, Qu Y, Zhao G, Ren Y: **Hepatitis B Virus Infection and Hepatitis G Virus Antibody Test in Healthy People**. *Chinese Journal of Public Health* 1997(4):34.

19. Lei Y, Zhao L, Lou X, Ji Y: **Hepatitis G Virus Antibody Assay in 749 Blood Donor**. *The Journal of Medical Theory and Practice* 1998, **11**(7):306.

20. Ren FR, Wang Y, Li H, Chen HS, Zhao HY: **Hepatitis G virus infection in screened Chinese blood donors**. *Vox sanguinis* 1998, **74**(1):51-52.

21. Ru H, Wen X, Liang W: **[Investigation of HGV infection in various populations in Guangxi]**. *Zhonghua shi yan he lin chuang bing du xue za zhi = Zhonghua shiyan he linchuang bingduxue zazhi = Chinese journal of experimental and clinical virology* 1998, **12**(4):336-339.

22. Yang M, Chen Z, Ding G: **Detection of Anti-HGV IgG in Serum of Some Population in Chengdu Area**. *Journal of Sichuan Continuing Education College of Medical Sciences* 1998(3):55.

23. Zhang Y, Zhang D, Xu Z: **Detection of Anti-HGV in Serum of Liver Disease Patients and Blood Donors and Its Significance**. *Modern Diagnosis & Treatment* 1998(2):15-16.

24. Bao Y, Sun H, Niu H, Li G, Yue J, He Z, Jiang L: **Study on Current Situation of Hepatitis G Virus in Partial Blood Donors in Urumqi City**. *Xinjiang Medical Journal* 1998, **28**(4):215-216.

25. Wei Q, Hu S, Luo T, Yang D: **Analysis of Serum HGV Antibody Test Results of Healthy Blood Donors in Hubei**. *Acta Universitatis Medicinae Tongji* 1998, **27**(6):486-487.

26. Xu Y, Han W: **Investigation on Hepatitis G Virus Infection among Healthy People and Blood Donors**. *People's Military Surgeon* 1998, **41**(2):98-99.

27. Fu Y, Lv L, Zhou B, Luo H: **Detection of Hepatitis G Virus RNA in High Risk Population in Guangdong Province by Reverse Transcription Polymerase Chain Reaction**. *The Journal of Practical Medicine* 1998, **14**(10):724-726.

28. Yang Y: **Analysis of Hepatitis G Virus Infection in General Population and Blood Donor**. *The Journal of Practical Medicine* 1998, **14**(12):884-885.

29. Song Y, Wang C, Kong X: **Preliminary Study on Hepatitis G Virus Infection in Tongling City**. *Journal Of Bengbu Medical College* 1998, **23**(4):261.

30. Ling B, Zhuang H, Li S, Yang L, Cui Y: **Analysis of Hepatitis G Infection in Different Populations and Hepatitis Patients in China**. *Chinese Journal of Public Health* 1998, **14**(3):145-146.

31. Duan H, Zou X, Teng B: **Blood Donor Anti-HGV Detection and Analysis**. *Journal of Third Military Medical University* 1998(6):62,67.

32. Liu J, Yu A, Chen Z, Liu C, Sun D, Yang Y, Ji F, Jia W, Xia H, Wang Y *et al*: **Investigation on Hepatitis G Virus Infection among Blood Donors**. *Disease Surveillance* 1998(13):3.

33. Jiang C, Zhen Y, Hou H, Li Y, Chen C, Li G, She J: **Serological epidemiological survey of hepatitis G in blood donors**. *Chinese Journal of Blood Transfusion* 1998, **11**(4):213-214.

34. Feng G, Du H, Yi R, Hou S, Yu J, Wu Y, Huang Y, Zhen C, Li G: **Research on Detection and Control of Blood Donor-related Hepatitis Index**. *Shaanxi Journal of Medical Laboratory Sciences* 1998(2):4-6.

35. Deng R, Liu Z: **Investigation of Hepatitis G Virus Antibodies in Blood Donors**. *Jiangxi Journal of Medical Laboratory Sciences* 1998, **16**(3):133.

36. Mao G, Fang J: **Investigation on Anti-hepatitis G Antibodies in Serum of Some Natural Population in Xiaoshan City**. *Journal of Radioimmunology* 1998(2):89-90.

37. Cong Y, Tan W, Chen G, Miao J, Zhang W, Tian R, Zhan M: **Detection of Hepatitis G Virus Infection in Different Clinical Hepatitis/liver Disease Patients in China by Reverse Transcription Nested Polymerase Chain Reaction**. *Chinese Journal of Experimental and Clinical Virology* 1998, **12**(2):173-175.

38. Yan L, Jiang Q, Cao Y, Li A, Zhu F, Fu Q: **Investigation on Hepatitis G Virus Infection in Paid Blood Donors**. *Zhejiang Journal of Preventive Medicine* 1998, **10**(12):718-719.

39. Wang Z, Wang J, Shui L, Fu H, Yan S: **Detection of hepatitis G virus antibody in 174 blood donors in Zhoushan City**. *Zhejiang Journal of Preventive Medicine* 1998, **10**(8):483.

40. Zhao G, Zhou Q, He Y, Huang P, Liu J: **Detection of HGV RNA in Blood Donor Mixed Samples by RT-nested PCR**. *China Blood Transfusion 1999 Annual Conference* 1999.

41. Chen X, Xuan M, Wu D: **[Study on hepatitis G virus infection]**. *Zhonghua liu xing bing xue za zhi = Zhonghua liuxingbingxue zazhi* 1999, **20**(2):85-87.

42. Song H, Dong H: **Analysis of Hepatitis G Virus Infection in Different Populations and Hepatitis Patients in Anhui Province**. *Progress in Microbiology and Immunology* 1999, **27**(4):42-44.

43. Huang C, Qin Y, Qin H, Huang Q, He Y, Qin X, Zhou Y, Huang W, Huang H: **Study on Hepatitis G Virus Infection in Different Populations in Bose Ethnic Region**. *Journal of Youjiang Medical College For Nationalities* 1999, **21**(3):370-372.

44. Liu Y, Sheng L, Liu X, Cai J, Hu W: **Serological Epidemiological Investigation of Blood Donor HGV Infection in Fengxian County**. *Shanghai Journal of Preventive Medicine* 1999, **11**(3):111-112.

45. Yao C, Zhou Y, Li G, Chen T, Huang Y, Peng W: **Analysis of Hepatitis G Virus Infection in Hepatocellular Carcinoma Patients and Blood Donors**. *Cancer* 1999, **18**(2):136-.

46. Zhang P, Zang R, Hu X, Xue T, Li S, Hu T: **Serological Surveillance and Analysis of Incidence of G-Hepatitis Virus**. *Chinese Journal of Frontier Health and Quarantine* 1999, **22**(1):5-7.

47. Chen X, Xuan M, Wu D, Yin Y, Zhou Y, Wan B, Wei Y, Fu C: **Study of Hepatitis G virus infection**. *Chinese Journal of Epidemiology* 1999, **20**(2):85-87.

48. Song Y, Pan E, Kong X: **Preliminary Investigation of Hepatitis G Virus Infection**. *Journal of Clinical Hepatology* 1999(01).

49. Zhou B, Xu H: **Application of Hepatitis G Virus Antibody Test in Blood Transfusion**. *Journal of Bengbu Medical College* 1999, **24**(3):193.

50. Ye D, Huang F, Hu Z, Zhen H, Wang X: **Study on Molecular Biology of Hepatitis G Virus from Blood Donors in Hefei Area**. *Journal of Clinical Transfusion and Laboratory Medicine* 1999(02):16-19.

51. Shen W, Wang C, Huang Y: **Investigation and Analysis of Hepatitis G Virus Infection in Different Populations in Henan Province**. *Chinese Journal of Health Laboratory Technology* 1999(02):32-33.

52. Li G, Liao J, Ma H, Liang Y, Yao J, Yao C, Su Y, Tang W, Chong Y, Chen Q: **Analysis of Prevalence and Homology of HGV in South China**. *Chinese Journal of Microbiology and Immunology* 1999, **19**(3):206-210.

53. Wang Y, Zhuang H, Zhang H, Wu X, Tj H, Li H: **Comparison of anti-HGV antibody enzyme-linked immunosorbent assay (EIA) and HGV PCR assay results**. *Chinese Journal of Public Health* 1999, **15**(4):330.

54. Yang Y: **Analysis of Hepatitis G Virus Infection in General Population and Blood Donor**. *Acta Academiae Medicinae Suzhou* 1999, **19**(1):51.

55. Yang Y, Li F, Wang Y, Zhang J, Zhu F, Yu Z, Zhao X, Yang D: **Detection of Serum TTV and Other Viral Nucleic Acids in Occupational Blood Donors in Wuhan**. *Journal of Practical Hepatology* 1999(04):202-203.

56. Zhao G, Xing P, Su D, Hu Z, Liu L, Ma H, Wang K: **Blood Donor Hepatitis G Virus Infection and Partial Gene Sequence Analysis**. *Journal of Henan Medical University* 1999, **34**(2):28-31.

57. Liu T, Han Y, Zhang Y: **Detection and Analysis of Hepatitis G Virus Nucleic Acid in Blood Donors and Hemodialysis Patients**. *Journal of Youjiang Medical College For Nationalities* 1999(04):184.

58. Wu F: **Detection of Hepatitis G Virus and Its Antibody in Blood Donors**. *Chinese Journal of Blood Transfusion* 1999, **12**(3):195.

59. Pan X, Wei L, Wu W, Zhang Y: **Detection and Analysis of Hepatitis G Virus Infection in Some Populations in Xuzhou Area**. *Acta Academiae Medicinae Xuzhou* 1999, **19**(4):262-264.

60. Wang Q, Du Z, Meng Q, Liu Y, Chi B: **Study on Hepatitis G Virus Infection by Reverse Transcriptase Chain Reaction**. *Journal of Clinical Hepatology* 1999, **15**(1):27-29.

61. Wang Z, Zhao G, Tang S, Li A: **Detection of Hepatitis G Infection in People in Zibo District of Shandong Province by RT-PCR**. *Practical Preventive Medicine* 1999, **6**(2):84-85.

62. Lin T, He X, Gao X, Xu W: **Investigation of HGV Infection in 808 Blood Donors**. *Journal of Clinical Transfusion and Laboratory Medicine* 2000(01):35.

63. Zhen Y, Liang M, Wang J: **[Detection of IgG antibody to hepatitis G virus by ELISA]**. *Zhonghua shi yan he lin chuang bing du xue za zhi = Zhonghua shiyan he linchuang bingduxue zazhi = Chinese journal of experimental and clinical virology* 2000, **14**(2):151-153.

64. Tian DY, Yang DF, Xia NS, Zhang ZG, Lei HB, Huang YC: **Serological prevalence and risk factor analysis of hepatitis G virus infection in Hubei Province of China**. *World journal of gastroenterology* 2000, **6**(4):585-587.

65. Liu J, Li Y: **Study on molecular epidemiology of hepatitis G virus infection in Chongqing**. *World Chinese Journal of Digestology* 2000, **8**(4):410-412.

66. Jia X, Lv Z, Xu P, Wang W, Liu D, Zhang Y: **Investigation of hepatitis G virus infection in different populations in Dalian**. *Chinese Journal of Public Health* 2000(06):49.

67. Zhang X, Song G, Sun F, Li C: **Serological Investigation of Hepatitis G Virus Infection**. *Chinese Journal of Nosocomiology* 2000(04):280.

68. Yang X, Li Y, Liu C, Hao L, Wu F: **Study on Transmission Pathway and Risk Factors of Hepatitis G**. *Chinese Journal of Public Health* 2000(06):64.

69. Li Y, Li R, Yang J, Gong J, Yang Y, Huang Y, Li G, Li Z, Huang Q, Nong Y: **Investigation on Hepatitis G Infection in Different Populations in Guangxi**. *Guangxi Journal of Preventive Medicine* 2000, **6**(1):9-11.

70. Xing P, Zhao G, Liu L, Zhang S: **Hepatitis G Virus Infection and Gene Variation in Henan Province**. *Henan Medical Research* 2000, **9**(4):300-303.

71. Zhou X, Xiong Y, Xie J, Gong J, Ding X: **Investigation on Hepatitis G Infection of Partial Blood Transfusion Workers in Jiangxi Province**. *Chinese Journal of Public Health* 2000(01):38.

72. Huang J, Mao M, Qin J: **Study on retesting of hepatitis B virus and blood transfusion-associated hepatitis**. *Journal of Guangxi Medical University* 2000(06):1047-.

73. Mei H, Yun Z: **Status and Analysis of Hepatitis G Virus Infection in Different Populations in Luoyang Region**. *Journal of Clinical Transfusion and Laboratory Medicine* 2000(03):26-27.

74. Li J, Li D, Guo J: **Investigation of Hepatitis G Virus Antibodies in Some Population in Qingyuan City**. *Occupation and Health* 2000(11):65.

75. Cao M, Shao X, Lan K, Li L, Zhang Z, Jiang S, Zhao W: **Serological Survey of Hepatitis G Virus in Some Populations in Shaanxi, Qinghai and Xinjiang Provinces**. *Medical Journal of National Defending Forces in Northwest China* 2000, **21**(3):192-193.

76. Song Y, Chen L, Pan E, Chen Y, Kong X: **Investigation on Hepatitis G Virus Infection in Specific Population in Tongling Area**. *Acta Universitatis Medicinalis Anhui* 2000, **35**(3):225-226.

77. Li K, Zhang L: **Preliminary Investigation on Hepatitis G Virus Infection among Blood Donors in Tongling Area**. *Labeled Immunoassays and Clinical Medicine* 2000, **7**(1):8-10.

78. Li F, Yang Y, Yu Z, Zhu F, Wang Y, Zhang J, Zhao X, Yang D: **Detection of Hepatitis-associated Virus and Other Hepatitis-associated Viruses in Serum of Blood Donors in Wuhan**. *Journal of Xianning Medical College* 2000(02):100-102.

79. Li L, Zhang X, Mu S, Li C, Li C: **Blood Donor HGV and HBV and HCV Coinfection in Xi'an**. *Journal of Fourth Military Medical University* 2000, **21**(2):146-147.

80. Wang M, Mao X: **Analysis of Hepatitis G in Blood of Blood Donors and Hepatitis C Patients**. *Journal of Radioimmunology* 2000, **13**(2):105-106.

81. Zhang L, Wen H, Zhao H: **A Control Study of Hepatitis G Virus Infection in Blood Donors**. *Journal of Shanxi Medical University* 2000, **31**(1):71-72.

82. Li X, Re X, Liang X, Li G, Wu M: **The status of hepatitis G virus infection in some high-risk groups in Xinjiang**. *Chinese Journal of Disease Control and Prevention* 2000, **4**(2):107-108.

83. Wang L, Zhang Q, Ling B, Xu W: **Hepatitis G virus infection and nucleic acid sequence determination in primary liver cancer and occupational blood donors**. *Acta Academiae Medicinae Shandong* 2000, **38**(2):161-162.

84. Feng X, Li D, Wang C: **Detection of HGV RNA in Different Populations by Anti-HGV and PCR with ELISA**. *Henan Journal of preventive Medicine* 2001(03):131-132.

85. Yan J, Chen LL, Luo YH, Mao YF, He M: **High frequencies of HGV and TTV infections in blood donors in Hangzhou**. *World journal of gastroenterology* 2001, **7**(5):637-641.

86. Liu C, Liu W, Pu S, Xu A: **Investigation on Hepatitis G Virus Infection among Blood Donors in Panzhihua City**. *Journal of Sichuan Continuing Education College of Medical Sciences* 2001, **20**(4):282.

87. Cheng W, Xing Y, Liu A, Wang W: **Analysis of HGV and TTV Detection in Uygur and Han Blood Donors**. *World JOurnal of Medicine Today* 2001, **2**(12):1046-1048.

88. Li X, Liu X, Kong J, Wang X: **Study on Hepatitis G Virus Infection in Blood Donors in Northern China**. *Chinese Journal of Experimental and Clinical Virology* 2001, **15**(3):2.

89. Liu C, Chen X, Yan J: **Survey of HGV and TTV Infection in Blood Donors in Zhejiang Province**. *Journal of Microbiology* 2001, **21**(4):28-29.

90. Li G, Ma HH, Yao CL, Chong YT, Yao JL, Lau GKK, Leung YK, Tang WH: **Prevalence of hepatitis G virus infection and homology of different viral strains in Southern China**. *World journal of gastroenterology* 2002, **8**(6):1081-1087.

91. Han X, Fang J, Jin C: **Investigation of Hepatitis G Virus Infection in Yanbian Area**. *Journal of Medical Science Yanbian University* 2002, **25**(3):177-179.

92. Xi Z, Zhang Y, Peng L, Jia X, Zhang W, Zhang Z, Yuan H: **Analysis of Hepatitis G Virus Infection in Professional Blood Donors**. *Progress in Microbiology and Immunology* 2002, **30**(3):26-28.

93. Wang G, Li X, Sun R, Tian H, Tian M: **Analysis of Serum Enzymes and Hepatitis Serum Markers in Blood Donors**. *Shanghai Journal of Medical Laboratory Sciences* 2003(04):230-231.

94. Bai L, Wang L: **Detection of Hepatitis C Virus and Hepatitis G Virus RNA in "Healthy" Professional Blood Donors**. *Chinese Journal of Clinical Medicine (Guangzhou)* 2003(65):139-140.

95. Wang X, Tang W, Zhang D, Ji Y: **Preliminary investigation on the infection status of TTV, HGV and HCMV in several types of population in Nantong City**. *Journal of Tropical Medicine* 2003, **3**(2):181-183.

96. Wu J, Jiang Y, Yan X: **Detection of Hepatitis G Virus Infection by Nested PCR Method**. *International Journal of Laboratory Medicine* 2006, **27**(8):673-676.

97. Huang X, Pang D: **Analysis of Hepatitis G Virus Infection among Different Populations in Nanning City**. *Journal of Youjiang Medical College For Nationalities* 2009, **31**(2):260-261.

98. Jia X, Huang X, Lv Z: **Investigation of GBV-C/HGV Infection in Different Populations in Dalian City in 2009**. *Preventive Medicine Tribune* 2011, **17**(9):793-794.

99. Xiao W, Lin F, Sun P, Ma L, Li C: **Detection of GB virus C/hepatitis G markers in Chinese voluntary blood donors**. *Brazilian Journal of Infectious Diseases* 2014, **18**(3):352-353.

100. Yu H, Liang W, Liu Y, Guo J, Deng K, Lin X: **Study on TTV Infection and Over-infection with HBV, HCV and HGV among Non-Requited Blood Donors in Yao Nationality**. *Chinese Journal of Blood Transfusion* 2014(01):36-38.

101. Jin Y, Xu J, Cao Y, Fang Z, Dong L, Jiang N, Wang L: **Seroepidemiological investigation of hepatitis G virus infection in unpaid blood donors in Suzhou**. *International Journal of Laboratory Medicine* 2016, **37**(13):1882-1883.

102. Shao C, Zhuo H, Luo Q: **Investigation on hepatitis g infection in voluntary blood donors in Beijing area**. *Vox sanguinis* 2017, **112**:92.
